# Antiproliferative Effects of DNA Methyltransferase 3B Depletion Are Not Associated with DNA Demethylation

**DOI:** 10.1371/journal.pone.0036125

**Published:** 2012-05-01

**Authors:** Sabine Hagemann, Dirk Kuck, Carlo Stresemann, Florian Prinz, Bodo Brueckner, Cora Mund, Dominik Mumberg, Anette Sommer

**Affiliations:** 1 Division of Epigenetics, DKFZ-ZMBH Alliance, German Cancer Research Center, Heidelberg, Germany; 2 Global Drug Discovery, Bayer Healthcare Pharmaceuticals, Berlin, Germany; Technische Universität München, Germany

## Abstract

Silencing of genes by hypermethylation contributes to cancer progression and has been shown to occur with increased frequency at specific genomic loci. However, the precise mechanisms underlying the establishment and maintenance of aberrant methylation marks are still elusive. The de novo DNA methyltransferase 3B (DNMT3B) has been suggested to play an important role in the generation of cancer-specific methylation patterns. Previous studies have shown that a reduction of DNMT3B protein levels induces antiproliferative effects in cancer cells that were attributed to the demethylation and reactivation of tumor suppressor genes. However, methylation changes have not been analyzed in detail yet. Using RNA interference we reduced DNMT3B protein levels in colon cancer cell lines. Our results confirm that depletion of DNMT3B specifically reduced the proliferation rate of DNMT3B-overexpressing colon cancer cell lines. However, genome-scale DNA methylation profiling failed to reveal methylation changes at putative DNMT3B target genes, even in the complete absence of DNMT3B. These results show that DNMT3B is dispensable for the maintenance of aberrant DNA methylation patterns in human colon cancer cells and they have important implications for the development of targeted DNA methyltransferase inhibitors as epigenetic cancer drugs.

## Introduction

Epigenetic silencing of various genes by aberrant promoter hypermethylation is a common feature of human cancer cells [1]–[3]. Increasing evidence suggests that the establishment of this hypermethylation phenotype is a directed process with specific genes becoming preferentially methylated and inactivated in many different cancer types. Most notably, a CpG island methylator phenotype (CIMP) has been described, based on the methylation status of defined marker genes [4]. CIMP is characterized by widespread tumor-specific CpG island methylation and has been reported in several tumor types using different methods and different marker genes [4], [5].

The mechanisms for the establishment and the maintenance of gene-specific hypermethylation during cancer development have not been fully elucidated yet. However, several candidate factors have been identified which might play an important role in recruiting DNA methyltransferases to specific genomic loci in cancer cells. Therefore, dysregulation of recruiting factors or alterations of gene-specific chromatin modifications involved in recruitment could potentially lead to a hypermethylation phenotype in cancer. In agreement with this notion, it has been shown that genes that are associated with components of the Polycomb Repressive Complex 2 (PRC2) in embryonic stem (ES) cells are frequently hypermethylated in cancer [6]–[8]. Additionally, increased activity of DNA methyltransferases (DNMTs) in cancer cells could potentially lead to aberrant de novo methylation.

In human cells, DNA methylation is catalyzed by DNMT1, DNMT3A and DNMT3B [9]. During DNA replication, the so-called maintenance methyltransferase DNMT1 methylates hemimethylated DNA by copying methylation marks from the parental DNA strand to the newly synthesized daughter strand [10]. DNMT3A and DNMT3B enzymes preferentially methylate unmethylated DNA and are therefore denoted as de novo methyltransferases [11]. It has been shown that overexpression of the de novo methyltransferase DNMT3B induces hypermethylation of specific genes and repetitive elements in HEK293T cells [12]. Moreover, transgenic expression of DNMT3B in mice resulted in gene-specific de novo methylation at various loci [13], [14]. Furthermore, DNMT3B expression increases during colorectal cancer progression and correlates positively with the methylation level of CIMP marker genes [15], [16]. These studies implicate the de novo methyltransferase DNMT3B in the establishment of gene-specific hypermethylation during cancer development and progression.

In line with these findings, DNMT3B has recently been proposed to act as a bona fide oncogene in human cancer cell lines by correlating DNMT3B gene amplification with resistance to DNA demethylating drugs [17]. These results further supported the notion that overexpression of DNMT3B may contribute to aberrant DNA methylation in cancer and thus suggest DNMT3B as a candidate target for drug development in oncology. However, only few studies have investigated the specific role of DNMT3B in the establishment and maintenance of aberrant hypermethylation patterns in cancer cells. A mild reduction in genomic methylation levels has been described in DNMT3B knockout cells [18]. Moreover, DNMT3B short-term knockdown by RNAi resulted in demethylation and reactivation of RASSF1A in A549 lung cancer cells [19] and in demethylation of APC, RARß, and RB1 gene promoters in PC3 prostate cancer cells [20]. Recently, differential effects in gene re-expression and invasive behavior after siRNA-mediated knockdown of DNMT3B or DNMT1 in breast cancer cells have been reported [21]. Importantly, however, none of the previous studies has systematically analyzed the impact of DNMT3B protein reduction in parallel on proliferation and on the global gene methylation pattern. In this study, we used Infinium methylation arrays to interrogate the methylation level of putative DNMT3B target genes in DNMT3B-overexpressing and in DNMT3B-deficient colon cancer cells. While our results show that DNMT3B knockdown impacts on the proliferation of colon cancer cells, they also demonstrate that the enzyme is dispensable for the hypermethylation phenotype in these cells.

## Materials and Methods

### Cell culture

Cell lines have been obtained from different providers: HCT-116, Caco-2, HT-29, SW-480 from the German Collection of Microorganisms and Cell Cultures (DSMZ, Germany); HCT-15 and SW-620 from the NCI 60-Panel; DLD-1 and WI-38 cells from the ATCC. Human non-tumorigenic hTERT-immortalized mammary epithelial cells with reduced levels of p53 (HMEC-T53) were generated at Bayer Healthcare Pharmaceuticals, Berlin, Germany (Ulbricht, U. et al. in preparation). HCT-116, Caco-2, and HT29 cells were grown in DMEM/Ham's F12 medium supplemented with L-glutamine (Biochrom) and 10% fetal calf serum (PAA). SW-480, HCT-15, SW-620, and DLD-1 cells were cultured in RPMI 1640 medium supplemented with L-glutamine (Biochrom) and 10% fetal calf serum (PAA). WI-38 cells were maintained in MEM Earle's medium supplemented with L-glutamine (Biochrom) and 10% fetal calf serum (PAA), and HMEC-T53 cells were grown in Mammary Epithelial Cell Growth Medium (Promocell: C-21010) and SupplementMix (Promocell: C-39115). HCT-116 DNMT3B knockout (3BKO), HCT-116 DNMT1 knockout (1KO) and HCT-116 DNMT1; DNMT3B double knockout (DKO) cells were kindly provided by Bert Vogelstein [18]. 3BKO, 1KO, and DKO cells were cultured in McCoy's 5A medium supplemented with 5% L-glutamine and 10% FCS (Invitrogen); the medium of 1KO cells was additionally supplemented with 50 µg/ml hygromycin (PAA). Early passages of all cell lines were used for the experiments.

### Expression analysis

Information on tissue-specific DNMT3B mRNA expression levels was obtained from the Array Northern database which is based on data of mRNA hybridized to Affymetrix arrays [22]. For obtaining mRNA expression levels of DNMT3B in various human normal organs, tissues and cancers, data from Affymetrix HGU133Plus2.0 arrays were retrieved. The corresponding probe set for DNMT3B expression was 220668_s_at. Gene expression values are shown as geometric mean values of the expression value on an arbitrary scale over all samples from a specific tissue type. For quantitative real-time PCR, RNA was extracted using the RNeasy Kit (Qiagen) and on-column genomic DNA digestion was performed using the RNase-Free DNase I Set (Qiagen). For qRT-PCR, 5 µg of RNA were reverse transcribed using the SuperScript III First-Strand Synthesis System (Invitrogen) according to the manufacturer's protocol. Subsequently, each cDNA sample was amplified in triplicate using the QuantiFast SYBRGreen PCR Kit and the QuantiTect primer assays Hs_DNMT3B_1_SG for DNMT3B and Hs_GAPDH_2_SG for GAPDH (Qiagen). The housekeeping gene Lamin B1 was amplified using the primers LAMIN_B1_fwd (5′-CTGGAAATGTTTGCATCGAAGA-3′) and LAMIN_B1_rev (5′-GCCTCCCATTGGTTGATCC-3′). The DNMT3B expression levels normalized to GAPDH or Lamin B1 were calculated with the deltaCt method.

For immunoblot analysis, cell pellets were resuspended in PBS (pH 7.4) containing Complete protease inhibitor cocktail (Roche) and 25 U Benzonase Nuclease (Novagen). Cells were disrupted using the Bioruptor Sonication system (Diagenode). Equal amounts of protein were separated on 10% SDS-polyacrylamide gels and transferred onto nitrocellulose membranes (Whatman). Proteins were detected using antibodies directed against DNMT3B (N-19, 1∶500, Santa Cruz Biotechnology) and β-actin (ab8226, 1∶10,000, Abcam). Horseradish peroxidase-conjugated secondary antibodies were visualized by enhanced chemiluminescence (ECL, Perkin-Elmer) and protein expression was quantified using the ImageJ software [23]. The signal intensity of DNMT3B was normalized to β-actin signal and the normalized signal values were transformed in a heatmap color code.

### DNMT3B RNAi knockdown

siRNA duplexes and non-targeting control siRNAs were obtained from Qiagen (DNMT3B siRNA#1, SI00092967; DNMT3B siRNA#2, SI00092974; DNMT3B siRNA#3, SI03038952; DNMT3B siRNA#4, SI03068240; DNMT3B siRNA#5, SI04987157; DNMT3B siRNA#6, SI04987164; non-targeting control siRNA, #1027280). We confirmed in silico that DNMT3B siRNAs target all mRNA isoforms of the active DNMT3B enzyme. 24 hours after cell plating, cells were transfected with 5 nM siRNAs for 72 hours using Lipofectamine RNAiMAX reagent (Invitrogen). Transfection was repeated every 24 hours to increase knockdown efficiency. For the generation of stable shDNMT3B clones, preselected shRNAs were cloned into the lentiviral pGT396-Puro vectors. The shRNA sequences of the forward oligonucleotides were as follows: sh_nontargeting: 5′-TTCTCCGAACGTGTCACGTTTCAAGAGAACGTGACACGTTCGGAGAA-3′, shDNMT3B#1: 5′-GACGGATGCCTAGAGTTTATTCAAGAGATAAACTCTAGGCATCCGTC-3′, shDNMT3B#2: 5′-TGGAGATGGAGACAGTTCATTCAAGAGATGAACTGTCTCCATCTCCA-3′. For lentivirus production and gene transfer, HCT-116 cells were grown in DMEM/Ham's F12 medium supplemented with L-glutamine and 10% fetal calf serum (PAA). Selection was performed in the presence of 1 µg/ml puromycin (Sigma). Lentiviruses were produced by co-transfecting the respective pGT396-Puro construct with lentiviral Packaging Mix (Invitrogen) into HEK-293FT cells (Invitrogen). Viral supernatants were collected and concentrated by ultracentrifugation (2 h, 50000× g). Viral titers were determined using a HIV p24 ELISA (Perkin Elmer). Transductions were carried out using p24 concentrations of 1 µg/ml for 6 h at 37°C.

### Cell viability and apoptosis analyses

Cell viability was measured in 96-well plates using the CellTiter-Glo assay (Promega) according to the manufacturer's instructions. The TECAN Infinite 200 microplate reader was used for detection of luminescence signals. For the quantitative detection of caspase 3/7 activities, the Caspase-Glo 3/7 Assay (Promega) was used according to the manufacturer's protocol. Assays were performed in 96-multiwell plates and analyzed by a TECAN Infinite 200 microplate reader. For the adjustment of cell numbers, cell viability was determined in parallel and used for normalization. Each sample was analyzed in quadruplicates; data are reported as mean ± SD.

### FACS analysis

Apoptotic and necrotic cells were analyzed with the FITC Annexin V Apoptosis Detection Kit I (BD Pharmingen). Cells were transfected with 20 nM of indicated siRNAs, 24 hours after cell plating. Transfection was repeated once after 24 hours to increase knockdown efficiency. Cells were washed with cold PBS and trypsinised 96 hours after the first transfection. After two washes with PBS, cells were stained according to the manufacturer's protocols. Samples were analyzed with a BD FACSCalibur™ flow cytometer. The fluorescence of 1×10^4^ cells was acquired and analyzed with CellQuest Pro software (Becton Dickinson). Annexin V-positive cells (as early marker of apoptosis) as well as Annexin V-positive and propidium iodide (PI)-positive cells (as early and late markers of apoptosis) were quantified.

### Array-based DNA methylation analysis

Array-based gene-specific DNA methylation analysis was performed using the Infinium HumanMethylation27 and HumanMethylation450 bead chip technology (Illumina) according to the manufacturer's instructions. Shortly, genomic DNA of different cell lines and siRNA-experiments (n = 1) was isolated with the DNeasy Blood and Tissue Kit (Qiagen) and deaminated using the EZ DNA Methylation Kit (Zymo Research). Results were analyzed using Illumina's BeadStudio software, version 3.1.3.0. The methylation status of specific cytosines is indicated by an average beta value (AVB) where 1 corresponds to complete methylation and 0 to no methylation. Signals of probes with *P*≥0.05 were excluded from the analysis. Loci were scored as hypermethylated if the AVB was greater than or equal to 0.8. Since changes in AVB greater than 0.2 can be detected with 95% statistical confidence, we used this value as a threshold to identify significant methylation changes in our analyses [24]. Infinium array methylation data are available in the ArrayExpress database (www.ebi.ac.uk/arrayexpress) under the accession number E-MTAB-719.

### Statistical analyses

Results were analyzed using using Illumina's BeadStudio software, version 3.1.3.0 and the R stats package, version 2.14.1 [25].

## Results

### DNMT3B is overexpressed in different cancer types

**Figure 1:**
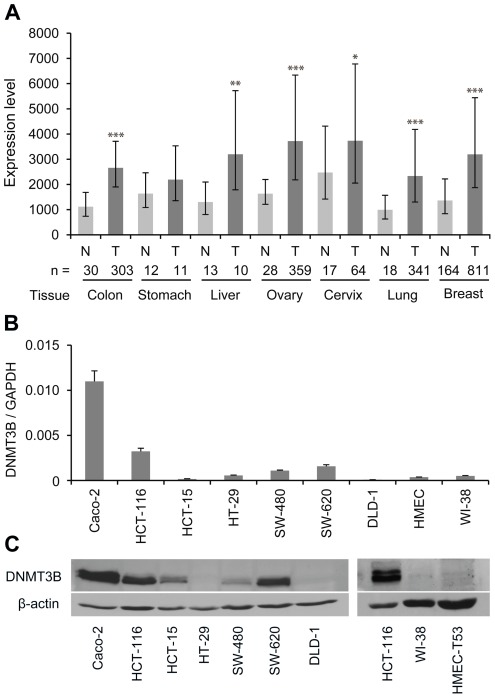
DNMT3B expression in human cancer tissue and colon cancer cell lines. (A) Array Northern analysis of DNMT3B mRNA expression on Affymetrix HGU133Plus2.0 arrays in tumor (T) tissue samples compared to normal (N) tissues of the same organ. n indicates the number of analyzed samples for the specified tissue type. Data are shown as mean±SD; *P<0.05 relative to normal tissue (N), **P<0.01 relative to normal tissue (N), ***P<0.001 relative to normal tissue (N), determined by student's t-test. (B) Quantitative real-time PCR analysis of DNMT3B mRNA expression in colon cancer cell lines and normal cells (HMEC-T53, WI-38). Expression values are means of triplicates and were calculated relative to GAPDH expression. Error bars represent standard errors. (C) Immunoblot of DNMT3B protein expression in lysates from various colon cancer cell lines and normal cells (HMEC-T53, WI-38). ß-actin was used as a loading control.

To determine the mRNA expression of DNMT3B in various human tissues and cancers, we analyzed the expression profiles in the Array Northern database [22], which is based on hybridization of RNA to the Affymetrix HGU133Plus2.0 array integrating internally and externally generated data. DNMT3B mRNA expression was elevated in lung, breast, cervix, ovarian, liver, gastric and colon cancers, respectively (Figure 1A). These findings indicate that DNMT3B mRNA is overexpressed in different cancer tissues and suggest a role of DNMT3B in the development or progression of various cancer types. As previous studies have implicated DNMT3B in colon cancer development [13]–[16], [26], we subsequently focused on characterizing the role of DNMT3B in colon cancer cell lines.

Using quantitative real-time PCR and immunoblotting, we screened a panel of human colon cancer cell lines for DNMT3B expression on the mRNA and protein level (Figure 1B, C). The results showed increased expression of DNMT3B in a subset of colon cancer cell lines, whereas normal human fibroblast WI-38 cells and human non-tumorigenic hTERT-immortalized mammary epithelial cells with reduced levels of p53 (HMEC-T53) showed only a very weak expression on the mRNA level and no detectable signal in immunoblots (Figure 1B, C). The DNMT3B mRNA expression levels were in good agreement with protein expression levels, with Caco-2 cells having the highest DNMT3B expression. HCT-116 and SW-620 cells expressed intermediate levels of DNMT3B, whereas HCT-15, SW-480, DLD-1, and HT-29 cell lines expressed low to non-detectable levels of DNMT3B. Due to the overexpression of DNMT3B in Caco-2 and HCT-116, these cells were selected as suitable models for further analyses.

### DNMT3B is required for survival of DNMT3B-overexpressing colon cancer cells

**Figure 2:**
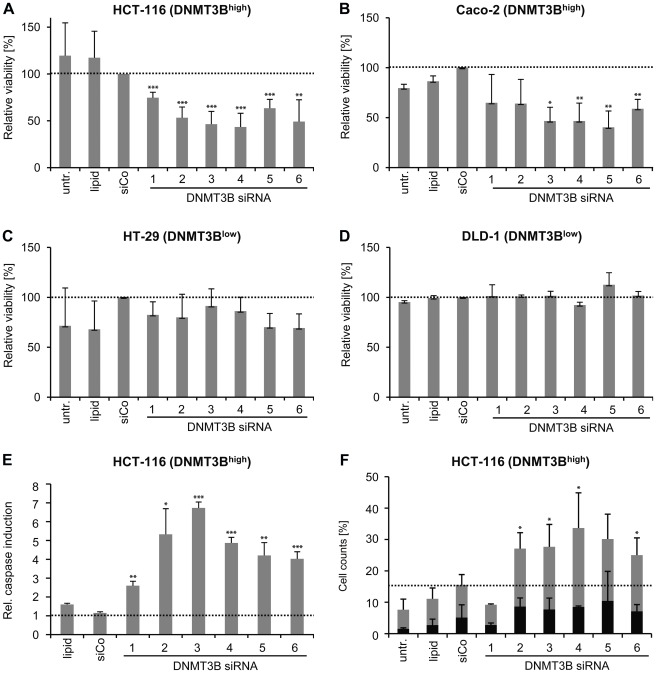
DNMT3B is required for survival of DNMT3B-overexpressing colon cancer cells. (A–D) Proliferation assays of colon cancer cells expressing high (A: HCT-116, B: Caco-2) or low (C: HT-29, D: DLD-1) levels of DNMT3B. Cell viability was measured 72 h after the first transfection (see Material and Methods) and normalized to cells transfected with non-targeting control siRNA (siCo); untr., untreated cells; lipid, transfection reagent control. Each sample was analyzed in quadruplicates; data are shown as mean ± SD, relative viability was used for normalization and set to 100% for siCo treated cells. (E) Caspase 3/7 activation assay of HCT-116 cells after siRNA knockdown of DNMT3B. Each sample was analyzed in quadruplicates; data are shown as mean±SD, relative viability was used for normalization and set to 100% for siCo treated cells. (F) FACS analysis of Annexin V-FITC and propidium iodide (PI) staining on HCT-116 cells after siRNA knockdown of DNMT3B is shown. Annexin V positive cells and the sum of Annexin V-positve and PI-positive cells were quantified. Values indicate means of three independent experiments. Error bars represent standard deviations. *P<0.05 relative to control siRNA (siCo), **P<0.01 relative to control siRNA (siCo), ***P<0.001 relative to control siRNA (siCo), determined by student's t-test prior to normalization.

To analyze whether DNMT3B-overexpressing cells require DNMT3B for proliferation and survival, we performed short-term siRNA knockdown experiments and subsequently analyzed cell proliferation with cell viability assays. To account for the fact that DNMT3B is expressed in many isoforms and to exclude off-target effects, we used six different siRNAs that specifically target different regions of the DNMT3B mRNA. Knockdown efficiency was analyzed by quantitative RT-PCR and confirmed to be in the range of 65% to 90% (Figure S1A–D). The knockdown of DNMT3B specifically reduced the viability of Caco-2 and HCT-116 (Figure 2A, B), the two cell lines that expressed high levels of DNMT3B, whereas the viability of cell lines with low DNMT3B expression levels such as HT-29 and DLD-1 was not affected (Figure 2C, D). After siRNA-mediated knockdown of DNMT3B in HCT-116 (knockdown efficiency 60% to 80%, Figure S1B), we also analyzed caspase 3/7 activation (Figure 2E) and Annexin V staining (Figure 2F) as markers for the induction of apoptosis. After DNMT3B knockdown in HCT-116 cells, caspase 3/7 was induced 2–4 fold (Figure 2E) and the number of apoptotic cells (both Annexin V as well as Annexin V- and PI-positive) was approximately doubled when compared to cells that were transfected with control siRNA (Figure 2F). Therefore both assays clearly showed apoptosis induction after transient knockdown of DNMT3B. In summary, our results demonstrate that high levels of DNMT3B are required for the proliferation of a subset of colon cancer cell lines, which is consistent with a proposed oncogenic function of the protein [17].

### DNMT3B overexpression does not correlate with CIMP in colon cancer cells

**Figure 3:**
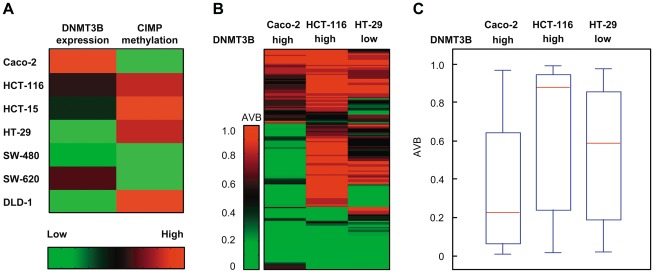
Endogenous level of DNMT3B protein expression does not correlate with CIMP status. (A) Comparison of DNMT3B expression and CIMP status. Red color indicates high levels of DNMT3B protein expression and high levels of CIMP marker methylation, green color indicates low levels of DNMT3B expression and low levels of CIMP marker methylation, respectively. Protein expression levels were derived from immunoblot analysis (Figure 1C) and methylation levels of the five CIMP marker genes CACNA1G, IGF2, NEUROG1, RUNX3, and SOCS1 in the cell lines were derived from [25]. (B) Heatmap of CG methylation at 174 loci representing 67 CIMP marker genes [25] in Caco-2, HCT-116, and HT-29 colon cancer cell lines. Average methylation was analyzed using Infinium 27 k methylation arrays. (C) Boxplots illustrate methylation levels of CIMP markers in Caco-2, HCT-116, and HT-29 cell lines. Lines in boxes denote medians, boxes the interquartile range, and whiskers the 2.5th and 97.5th percentiles, respectively. AVB, Average Beta value.

Transgenic overexpression of *Dnmt3b* in the murine intestine has been shown to cause DNA hypermethylation at various loci [13], [14]. In addition, it has been proposed that the hypermethylation of specific loci (CpG island methylator phenotype, CIMP) plays an important role in the formation of many cancer types, including colon cancer [15]. Moreover, it has recently been shown that DNMT3B overexpression is closely associated with the emergence of the CIMP phenotype in colorectal cancer [16]. We therefore analyzed the relationship between DNMT3B expression level and CIMP status using a classification based on a set of previously established CIMP markers (CACNA1G, IGF2, NEUROG1, RUNX3, SOCS1) [27]. Surprisingly, we could not detect any correlation between the two parameters in colon cancer cell lines. For example, CpG islands of the DNMT3B^high^ Caco-2 cells were completely unmethylated at the five CIMP markers, whereas CpG islands of the same genes were hypermethylated in the DNMT3B^low^ DLD-1 cells (Figure 3A). Since this classification was based on only five CIMP markers, we extended our analyses to an investigation of an enlarged set of CIMP markers in three selected cell lines, i.e. Caco-2 and HCT-116 cells with high DNMT3B expression and HT-29 cells with a very low DNMT3B expression level. We used Illumina Infinium 27 k methylation arrays to obtain genome-scale methylation profiles from all three cell lines and characterized the CIMP status using a set of 174 CpG sites representing 67 genes that had previously been identified as CIMP markers on the Illumina GoldenGate methylation platform [27]. Again, the methylation level of the promoter regions of these 67 genes did not correlate with DNMT3B expression levels in the three cell lines analyzed.

Caco-2 cells, expressing the highest level of DNMT3B protein, showed the lowest methylation of CIMP marker genes with only 23 out of 174 CpG loci (10%) being hypermethylated (AVB>0.8, see also Material and Methods), in contrast to HT-29 cells having 53 of 174 CIMP loci (30%) with an AVB>0.8. (Figure 3B). Infinium methylation analyses revealed the highest methylation of CIMP markers in HCT116 cells, as indicated by a total of 104 hypermethylated loci (60%) in the heat map (Figure 3B). Differences in the methylation of CIMP marker genes were also nicely reflected by the median methylation of CIMP loci in Caco-2, HCT-116, and HT-29 cells as illustrated in Figure 3C. Altogether, our data show no general association between a high protein expression level of DNMT3B and hypermethylation of CIMP markers in different colon cancer cell lines. This observation raises the possibility that the putative oncogenic function of DNMT3B overexpression may not exclusively be related to de novo DNA methylation of CIMP marker genes. In addition, different CI MP patterns may also be due to cancer cell line-specific effects.

### Depletion of DNMT3B has no effect on DNA methylation

**Figure 4:**
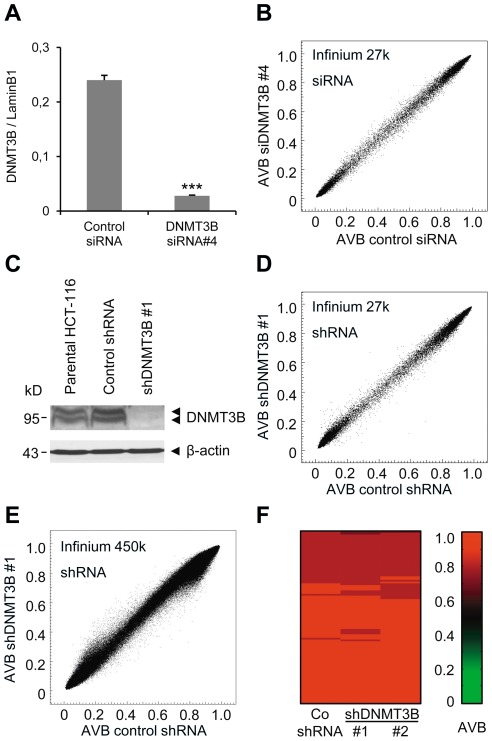
Efficient depletion of DNMT3B has no effect on DNA methylation. (A) DNMT3B mRNA levels were determined after siRNA knockdown with DNMT3B siRNA #4 by quantitative RT-PCR analysis. Expression values are means of triplicates and were calculated relative to Lamin B1 expression. Error bars represent standard errors. ***P<0.001 relative to control siRNA, determined by student's t-test prior to normalization. (B) Comparison of Infinium 27 k methylation profiles of HCT-116 cells transfected with DNMT3B siRNA #4 or control siRNA. (C) Efficient depletion of DNMT3B protein in stably shRNA-transduced HCT-116 cells. DNMT3B protein levels were determined by immunoblot analysis using ß-actin as a loading control. The double band presumably reflects the expression of two (or more) DNMT3B isoforms. (D) Comparison of Infinium 27 k methylation profiles between HCT-116 cells stably transduced with lentiviruses containing DNMT3B shRNA #1 and control cells transfected with a control shRNA. (E) Infinium 450 k methylation analysis comparing methylation profiles of HCT-116 cells transduced with DNMT3B-shRNA #1 and control cells transduced with a non-targeting control shRNA. (F) Heatmap based on the Infinium 450 k data, showing hypermethylated CIMP markers in HCT-116 cells stably transduced with shRNA #1 or #2 targeting DNMT3B or with a non-targeting control shRNA. AVB, Average Beta value.

To further analyze the role of DNMT3B in DNA methylation in colon cancer, we analyzed the effects of DNMT3B knockdown on the genomic DNA methylation pattern. For this purpose we used HCT-116 cells as a model, because this cell line is characterized by a high DNMT3B expression level and has a well-established hypermethylation phenotype. Efficient DNMT3B knockdown by four different siRNAs failed to reveal any obvious methylation changes on Infinium 27 k methylation arrays (Figure 4A, B and Figure S2 A–C). Moreover, even a long-term knockdown of DNMT3B using two different shRNA constructs did not result in significant changes of the DNA methylation pattern in HCT-116 cells (Figure 4C, D, and Figure S2D). Strong knockdown efficiency was confirmed on the mRNA or on the protein level (Figure S3A, B). Since Infinium 27 k arrays interrogate roughly 27,000 CpG loci located in promoter regions of about 14,000 annotated genes, we wondered whether DNMT3B may be involved in the methylation of genomic regions beyond those interrogated by the Infinium 27 k array. To test this hypothesis, we used Infinium 450 k methylation arrays, which - in addition to CpG loci in promoter regions - interrogate the methylation status of numerous CpG sites outside of CpG islands, addressing 99% of RefSeq genes with multiple probes per gene [28]. However, even with a substantially increased genome coverage we could not detect any obvious methylation changes after shRNA-mediated long-term knockdown of DNMT3B using 450 k methylation arrays. Only 37 isolated probes indicated a significant degree of hypomethylation, while 93 probes indicated hypermethylation, which corresponds to a negligible fraction of the analyzed genome (0.008% and 0.019%, respectively; Figure 4E). In light of the fact that virtually all genes are represented by >10 probes, these methylation changes probably reflect isolated experimental artifacts. In agreement with these observations, the methylation status of hypermethylated CIMP markers did not change after long-term knockdown of DNMT3B (Figure 4F). In summary, genome-wide methylation analyses demonstrate that DNMT3B does not play a significant role in maintaining methylation patterns of HCT-116 colon cancer cells.

### The hypermethylation phenotype is maintained in DNMT3B knockout cells

**Figure 5:**
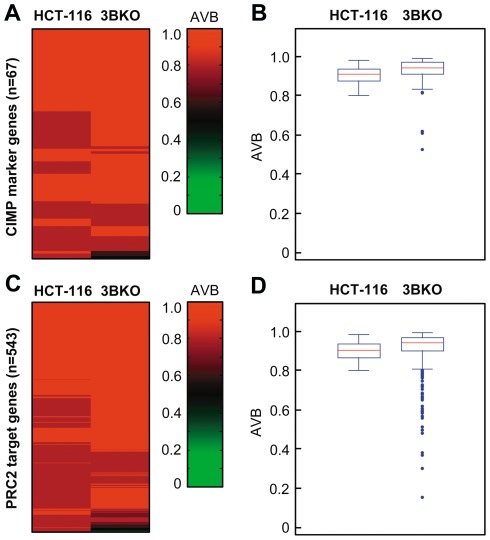
The hypermethylation phenotype is maintained in DNMT3B knockout cells. (A) Heatmap based on Infinium 27 k data illustrating the methylation levels of 67 CIMP marker genes in HCT-116 colon cancer cells and HCT-116 DNMT3B knockout cells (3BKO). (B) Boxplot illustrating methylation levels of 67 CIMP marker genes. Lines in boxes denote medians, boxes the interquartile range, and whiskers the 2.5th and 97.5th percentiles, respectively. (C) Heatmap based on Infinium 27 k data illustrating the methylation levels of 543 hypermethylated PRC2 target genes in HCT-116 colon cancer cells and HCT-116 DNMT3B knockout cells (3BKO). (D) Boxplot illustrating methylation levels of PRC2 target genes. AVB, Average Beta value.

Since RNAi-mediated knockdown does not completely deplete the DNMT3B protein from the cell, we wanted to rule out that residual amounts of DNMT3B protein might be sufficient to maintain CpG hypermethylation in HCT-116 cells. To address this question, we analyzed the methylation pattern of HCT-116 DNMT3B knockout cells [18]. An initial analysis of these cells at three defined loci had already suggested that loss of DNMT3B has only a comparably small effect on the DNA methylation pattern [18]. Our Infinium 27 k methylation data confirmed these results and also showed that the methylation levels of 67 CIMP marker gene promoters remained high in DNMT3B knockout cells (Figure 5A, B). Similar findings were also obtained when we investigated the effect of DNMT3B knockout on 543 hypermethylated PRC2 target genes. These genes are frequently de novo methylated in human cancers, including colon cancer [6]–[8]. However, also hypermethylated PRC2 target genes were not significantly demethylated in DNMT3B knockout cells (Figure 5C, D). Altogether, the data of the DNMT3B knockout cells confirm our previous results showing that DNMT3B is not required for maintaining the hypermethylated status of cancer-related genes in HCT-116 cells.

### Efficient demethylation of CIMP markers in DNMT1; DNMT3B double knockout cells

**Figure 6:**
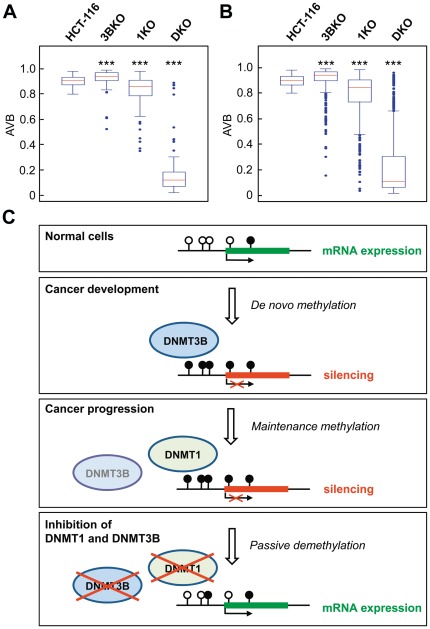
The hypermethylation phenotype is reversed in HCT-116 DNMT1; DNMT3B double knockout cells. (A, B) Boxplots illustrating the methylation levels of CIMP (A) and PRC2 (B) marker genes in parental HCT-116 cells (HCT-116), HCT-116 cells lacking DNMT3B (3BKO), HCT-116 cells with a hypomorphic DNMT1 allele (1KO), and HCT-116 DNMT1; DNMT3B double knockout cells (DKO) on Infinium 27 k methylation arrays. Lines in boxes denote medians, boxes the interquartile range, and whiskers the 2.5th and 97.5th percentiles. ***P<0.001 relative to parental HCT116 cells, determined by a Wilcoxon rank sum test. (C) Model for the role of DNMT3B during cancer development and progression. In normal cells, CpG islands are generally unmethylated and the corresponding genes are actively transcribed. During cancer development, certain regions become methylated by de novo methyltransferases such as DNMT3A and DNMT3B. Once the hypermethylation phenotype is established, the methylation pattern is maintained by the maintenance methyltransferase DNMT1 and becomes independent of DNMT3 enzymes. Only a strong reduction of overall methyltransferase activity causes the demethylation of hypermethylated cancer genes and inhibits the re-establishment by de novo methyltransferases.

Based on the analysis of both, global methylation levels and methylation of selected loci, it has been suggested that the combined genetic disruption of DNMT3B and DNMT1 causes a pronounced loss of DNA methylation [18], [29]. We therefore extended our analysis to include methylation profiles from HCT-116 double knockout cells (DKO) where both, the DNMT1 and the DNMT3B locus, had been targeted, as well as methylation profiles from DNMT3B-knockout (3BKO) HCT-116 cells and DNMT1 (1KO) HCT-116 cells with a hypomorphic DNMT1 allele [18]. The results from DKO cells showed a strong demethylation which encompassed the majority of the hypermethylated CIMP markers (Figure 6A). This pronounced effect was not observed in HCT-116 cells with a reduced activity of DNMT1, which is probably related to the hypomorphic nature of the mutant DNMT1 allele [30], [31]. We also observed a similar result for hypermethylated PRC2 target genes which again remained largely hypermethylated in DNMT1 hypomorphic cells but became extensively demethylated in DKO cells (Figure 6B). Together, these results further indicate that a combined inhibition of DNMT3B and DNMT1 is particularly effective for the demethylation of genes aberrantly hypermethylated in cancer.

## Discussion

The potential oncogenic functions of DNA methyltransferases represent a topic of considerable scientific interest and debate. While it is widely accepted that aberrant DNA methylation patterns represent a common hallmark of human cancers, it has been surprisingly difficult to attribute a corresponding functional significance to human DNA methyltransferase enzymes. A landmark study in the field has shown that reduced activity of DNMT1 suppresses intestinal tumorigenesis in a mouse colon cancer model [32]]. However, DNMT1 is also required for normal cellular function, e.g. for the inheritance of normal methylation marks during S-phase [33], [34] and therefore unlikely to play a major cancer-specific role.

DNMT3A and DNMT3B are two de novo DNA methyltransferases with potentially more specialized functions in the establishment of DNA methylation patterns during early stages of development [35], [36]. The co-ocurrence of DNMT3B overexpression and promoter hypermethylation observed in many human cancers suggested that DNMT3B-mediated de novo methylation might be functionally relevant for tumor development. In agreement with this notion, overexpression of DNMT3B has been shown to promote intestinal tumorigenesis in mouse models [13].

Our results suggest that DNMT3B is required for maintaining proliferation and for preventing apoptosis in cancer cell lines with a high level of endogenous DNMT3B. This finding is consistent with previous studies showing that depletion of DNMT3B induced apoptosis in cancer but not in normal cells in vitro [19], [20]. Although it was assumed that these effects would be caused by demethylation of DNMT3B target genes, this hypothesis was not confirmed experimentally. Our data indicate that the depletion of DNMT3B - while inhibiting proliferation and inducting apoptosis - does not induce any significant DNA methylation changes in established cancer cell lines. Even the complete absence of DNMT3B in 3BKO cells did not affect the hypermethylation phenotype of HCT-116 colon cancer cells, indicating that the observed antiproliferative effects of DNMT3B depletion are not caused by DNA demethylation of putative DNMT3B target genes. As we used high-content Infinium 27 k and 450 k methylation arrays that interrogate the methylation status of nearly all annotated genes in the human genome with multiple probes per gene, we cannot exclude that the knockdown of DNMT3B may effect the methylation of CpG loci that are not addressed by the array. However, our results clearly indicate that the CpG island methylator phenotype (CIMP) which has been established using the same array-based methods before [27] is not affected by depletion of DNMT3B in established colon cancer cell lines in vitro.

The effect of overexpression of DNMT3B7, a C-terminally truncated, catalytically inactive splice variant of DNMT3B, has recently been analyzed in a mouse model of B cell lymphoma [37]. Overexpression of DNMT3B7 in Eμ-myc transgenic mice induced growth of mediastinal lymphomas accompanied by increased genomic instability, a global increase of 5-methylcytosine levels and perturbations of specific DNA methylation patterns, where both an increase and decrease of methylation at certain CpG islands with high inter-tumor variability has been observed [37]. Similarly, also a conditional knockout of DNMT3B in a mouse model of myc-induced T-cell lymphomagenesis accelerated lymphomagenesis by increasing proliferation [38]. On the molecular level, DNMT3B-deficient T-cell lymphomas had increased genomic instability, a global decrease of 5-methylcytosine levels, e.g. on short interspersed nuclear elements (SINE). Analysis of global genome methylation showed that in lymphomas of DNMT3B knockout mice hypomethylation and to a four-fold lesser extent also hypermethylation occurred suggesting that DNMT3B seems to play a role in hypomethylation and hypermethylation of intragenic regions and promoters [38].

Taken together, expression of a catalytically inactive DNMT3B variant or a conditional knockout promote tumorigenesis in myc-driven murine models of lymphomagenesis, i.e. in cells of the hematopoietic lineage with concomitant perturbations of DNA methylation patterns. Besides differences in the response of epithelial versus hematological cells towards overexpression or knockdown of DNMT3B these data also indicate that murine hematological tumor model as well as developmental models [35], [36] have a higher level of plasticity with regard to alterations of DNA methylation than established cancer cells in vitro such as the HCT-116 colon cancer cells analyzed in our siRNA and shRNA knockdown studies. Lack of alteration of DNA methylation after DNMT3B siRNA or shRNA knockdown may therefore be limited to the in vitro models and “differentiated” cancer cells with already altered global methylation patterns. Our data does clearly not exclude potential effects on “cancer stem cell”-like cell populations, which have potentially still more plasticity in their DNA-methylation pattern.

The observed resistance to demethylation at these loci could be explained by redundant activities in the establishment and maintenance of DNA methylation by DNMT3A and DNMT1, respectively, being capable of activity compensating for the loss of DNMT3B in human cancer cells in vitro. However, this redundancy would not include proliferation control and survival. As such, our experiments clearly uncouple alteration of gene-specific de novo DNA methyltransferase activity from proliferation inhibition and apoptosis induction after knockdown of DNMT3B protein.

The molecular mechanisms regulating DNMT3B-dependent cell proliferation remain to be determined. In this context, it should be noted that DNMT3B interacts with various proteins, such as histone deacetylases, chromatin remodeling enzymes, transcriptional regulators, sumoylating and ubiquitinating proteins, and factors which play important roles in mitosis [39]–[42]. RNAi interference depletes DNMT3B proein levels from cells and may induce disruption of protein complexes which contain DNMT3B.

Thus, the alteration of gene activity, e.g. by released DNMT3B interaction partners, by methylation-independent mechanisms may cause changes in cellular proliferation after depletion of DNMT3B. Similar conclusions have been drawn from experiments using cell lines with experimentally reduced levels of DNMT1 protein [43], [44]. These data suggest that DNMT3B may not exclusively be involved in DNA methylation in established cancer cell lines but might also regulate other cellular processes by modulating the activity or localization of interacting enzymes.

Based on these observations we developed a model to illustrate the potential function of DNMT3B in cancer development (Figure 6C): In normal cells, CpG islands are generally unmethylated and the corresponding genes are actively transcribed. During cancer development, certain regions become methylated by the de novo methyltransferase DNMT3B. Once the hypermethylation phenotype is established, the maintenance methyltransferase activity of DNMT1 seems to be sufficient to maintain the aberrant methylation pattern. Therefore, already established hypermethylation patterns in cancer cells may be independent of the de novo methylation function of DNMT3B. This model is consistent with recent data from a mouse model showing that induced aberrant DNA methylation, after overexpression of Dnmt3b, is maintained in the absence of continuous Dnmt3b expression [14].

Importantly, our results are also relevant for optimizing demethylation efficacy during epigenetic therapy. Established nucleoside DNMT inhibitors like 5-azacytidine and decitabine are characterized by low specificity and substantial drug-dependent cytotoxicity. This necessitates the development of new, improved DNMT inhibitors, with the goal to reverse the CIMP pattern in e.g.colon cancer cells, to avoid the undesirable side effects in clinical applications. While our data suggest that targeting of DNMT1 alone is not sufficient for the demethylation of CIMP markers, this observation can probably be explained by the hypomorphic nature of the mutant DNMT1 allele, which still possesses significant residual enzymatic activity [30], [31]. Moreover,our data also suggest that also specifically targeting of DNM3B alone is not sufficient for the demethylation of CIMP markers. Based on our model, a dual specific DNMT1 and DNMT3B inhibitor would be the most efficient approach to reverse the hypermethylation phenotype in colon cancer cells. Inhibition of DNMT1 is crucial for disrupting the aberrant methylation at tumor-relevant genes and inhibition of DNMT3B is probably needed to interfere with de novo methylation.

## Supporting Information

Figure S1 **Analysis of DNMT3B knockdown for cell viability and apoptosis assays.** (A–D). Knockdown efficiency for cell viability and caspase assays was analyzed by quantitative RT-PCR in (A) Caco-2, (B) HCT-116, (C) HT-29, and (D) DLD-1 cells. Expression values are means of triplicates and were calculated relative to Lamin B1 expression. Error bars represent standard errors. Normalized expression values in control siRNA transfected cells were set as 1.0.
(TIF)

Figure S2 **Infinium 27 k methylation analysis of HCT-116 cells.** (A–C) Comparison of Infinium 27 k methylation profiles between HCT-116 cells transfected with either DNMT3B siRNA #3, #5, or #6, and HCT-116 cells transfected with control siRNAs, respectively. (D) Comparison of Infinium 27 k methylation profiles between HCT-116 cells stably transduced with lentiviruses containing DNMT3B shRNA #2 or transduced with control shRNA.
(TIF)

Figure S3 **Analysis of DNMT3B knockdown for Infinium methylation arrays.** (A) 72 h after transfection with the indicated siRNAs, DNMT3B mRNA levels were determined by quantitative RT-PCR analysis. Expression values are means of triplicates and were calculated relative to Lamin B1 expression. Error bars represent standard errors. Normalized expression values in HCT-116 control siRNA transfected cells were set as 100%. (B) Efficient depletion of DNMT3B protein in stably shRNA-transduced HCT-116 cells. DNMT3B protein levels were determined by immunoblot analysis using ß-actin as a loading control. The double band presumably reflects the expression of two (or more) DNMT3B isoforms.
(TIF)
