# Supplementary figures and images for: Antiproliferative Effects of DNA Methyltransferase 3B Depletion Are Not Associated with DNA Demethylation
Source: PLoS One. 2012 May 1;7(5):e36125. doi: 10.1371/journal.pone.0036125 (PMC3341356; doi:10.1371/journal.pone.0036125)

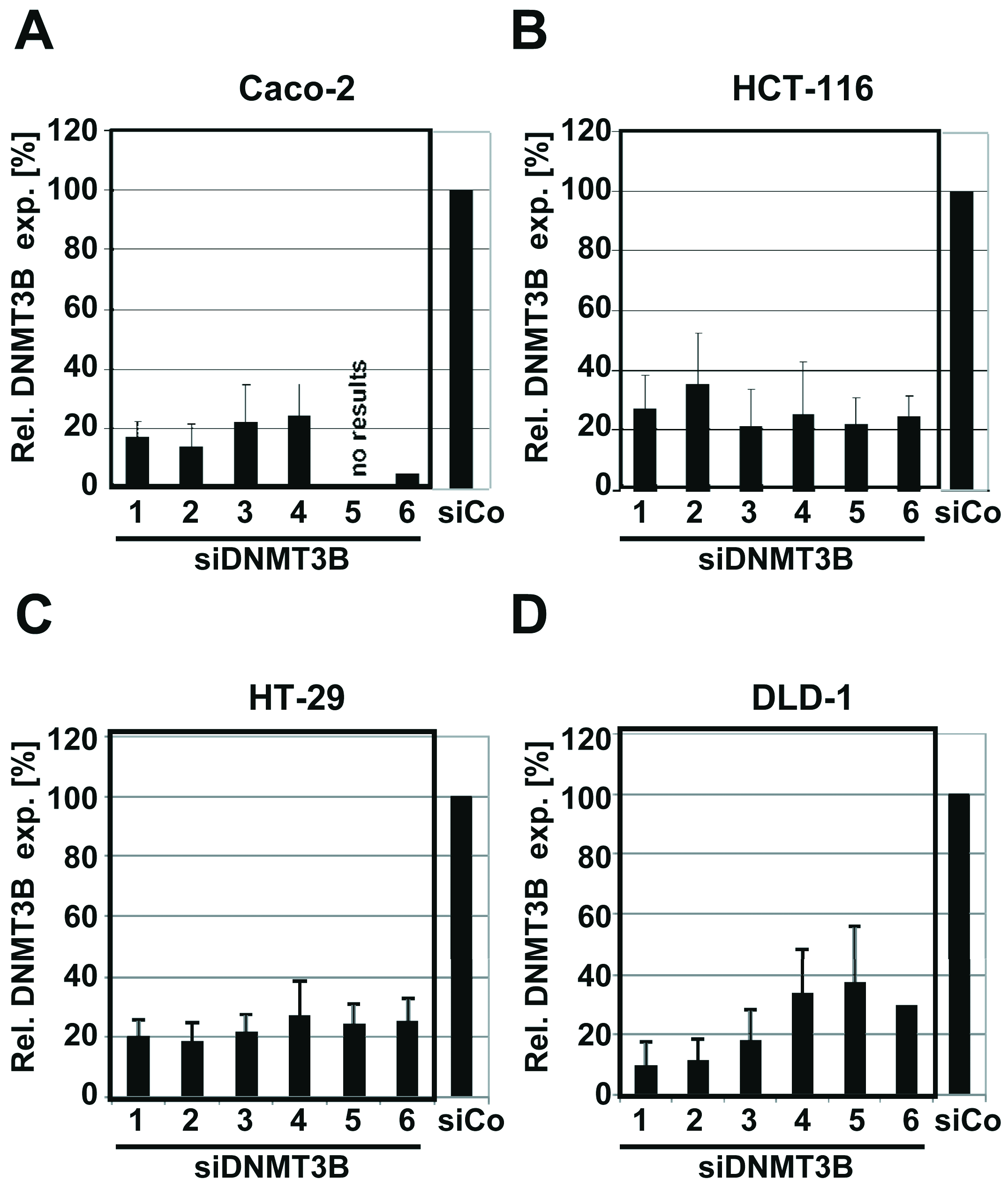

Supplement: Figure S1 — Analysis of DNMT3B knockdown for cell viability and apoptosis assays. (A–D). Knockdown efficiency for cell viability and caspase assays was analyzed by quantitative RT-PCR in (A) Caco-2, (B) HCT-116, (C) HT-29, and (D) DLD-1 cells. Expression values are means of triplicates and were calculated relative to Lamin B1 expression. Error bars represent standard errors. Normalized expression values in control siRNA transfected cells were set as 1.0. (TIF) [file pone.0036125.s001.tif]

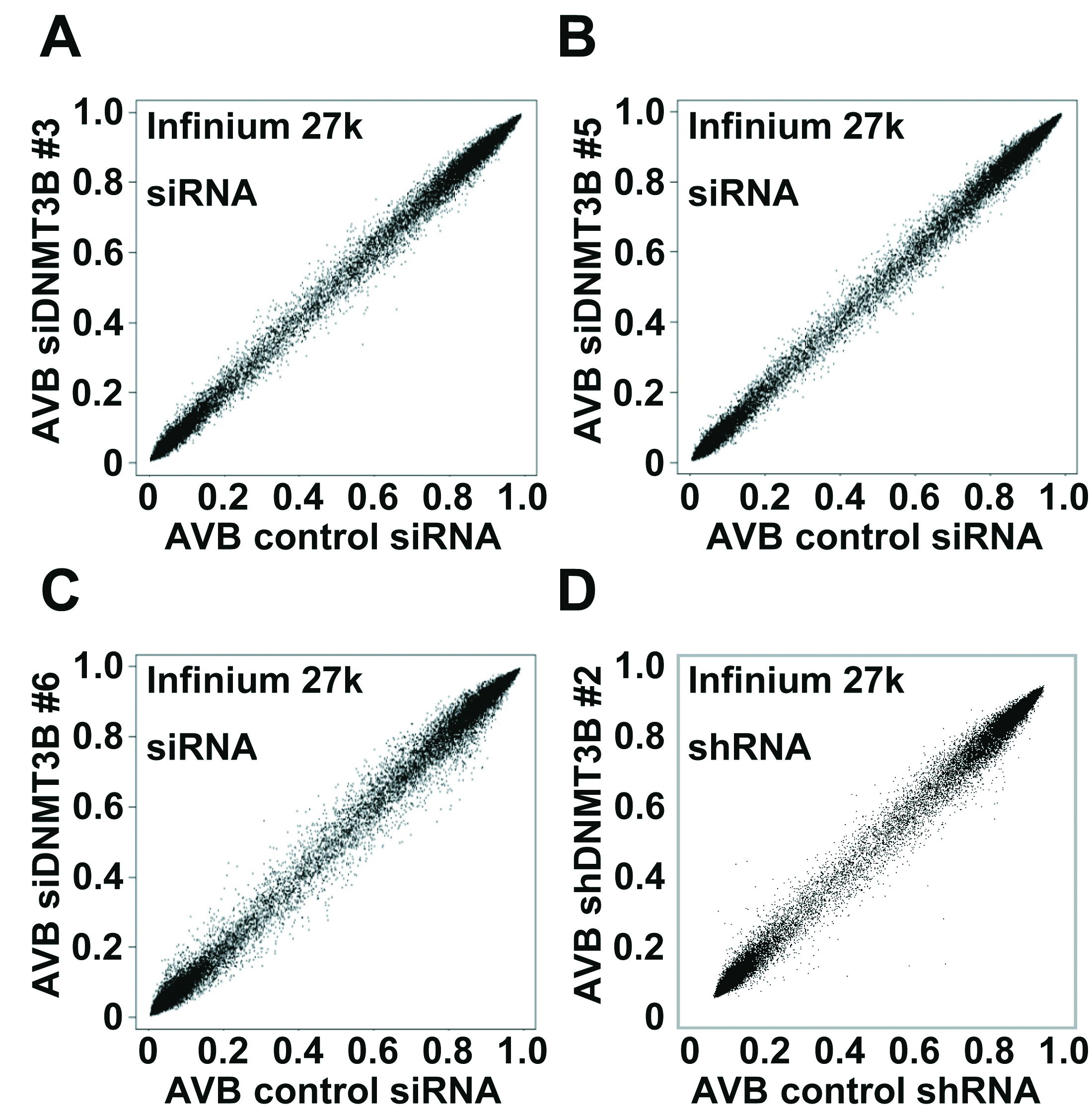

Supplement: Figure S2 — Infinium 27 k methylation analysis of HCT-116 cells. (A–C) Comparison of Infinium 27 k methylation profiles between HCT-116 cells transfected with either DNMT3B siRNA #3, #5, or #6, and HCT-116 cells transfected with control siRNAs, respectively. (D) Comparison of Infinium 27 k methylation profiles between HCT-116 cells stably transduced with lentiviruses containing DNMT3B shRNA #2 or transduced with control shRNA. (TIF) [file pone.0036125.s002.tif]

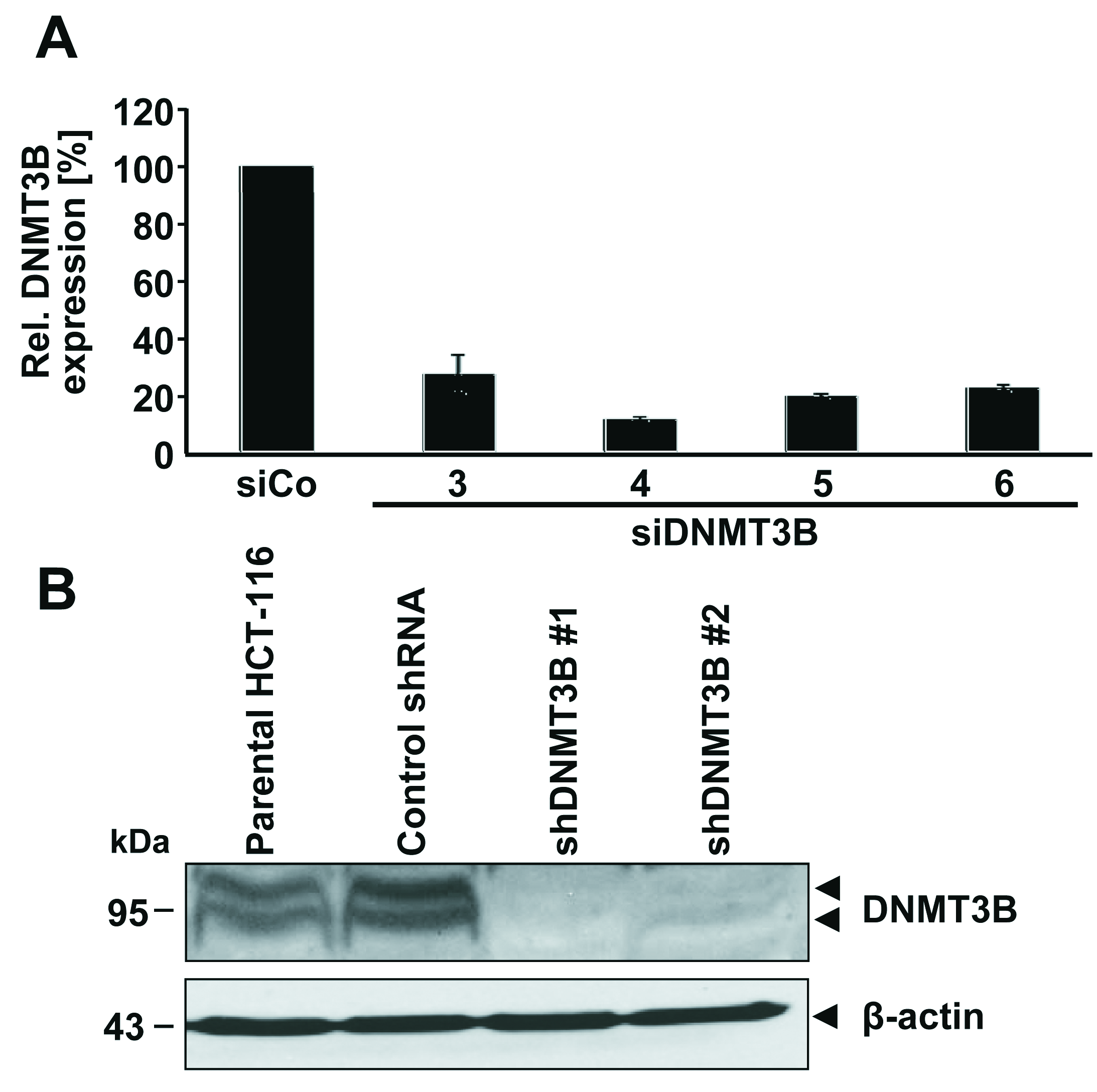

Supplement: Figure S3 — Analysis of DNMT3B knockdown for Infinium methylation arrays. (A) 72 h after transfection with the indicated siRNAs, DNMT3B mRNA levels were determined by quantitative RT-PCR analysis. Expression values are means of triplicates and were calculated relative to Lamin B1 expression. Error bars represent standard errors. Normalized expression values in HCT-116 control siRNA transfected cells were set as 100%. (B) Efficient depletion of DNMT3B protein in stably shRNA-transduced HCT-116 cells. DNMT3B protein levels were determined by immunoblot analysis using ß-actin as a loading control. The double band presumably reflects the expression of two (or more) DNMT3B isoforms. (TIF) [file pone.0036125.s003.tif]
